# Facile synthesis of two diastereomeric indolizidines corresponding to the postulated structure of alkaloid 5,9*E*-259B from a Bufonid toad (*Melanophryniscus*)

**DOI:** 10.1186/1860-5397-4-6

**Published:** 2008-01-21

**Authors:** Angela Nelson, H Martin Garraffo, Thomas F Spande, John W Daly, Paul J Stevenson

**Affiliations:** 1 School of Chemistry and Chemical Engineering, Queens University, Belfast, BT9 5AG, Northern Ireland; 2 Laboratory of Bioorganic Chemistry, National Institute of Diabetes and Digestive and Kidney Diseases, National Institutes of Health, Bethesda, MD 20892-0820, USA

## Abstract

A short synthesis of the postulated structure for indolizidine alkaloid **259B** with the hydrogens at C5 and C9 *entgegen* has been achieved with complete control of stereochemistry at C5. Both diastereoisomers at C8 were obtained, but neither proved to be the natural product. The comparison of the mass and FTIR spectral properties of the synthetic compounds to those of the natural material strongly suggest that the gross structure is correct and that the difference may be a branch in the C5 alkyl side-chain. The GC-retention times of the two synthetic compounds were markedly longer than that of the natural 5,9*E*-**259B**.

## Background

**Figure 1:**
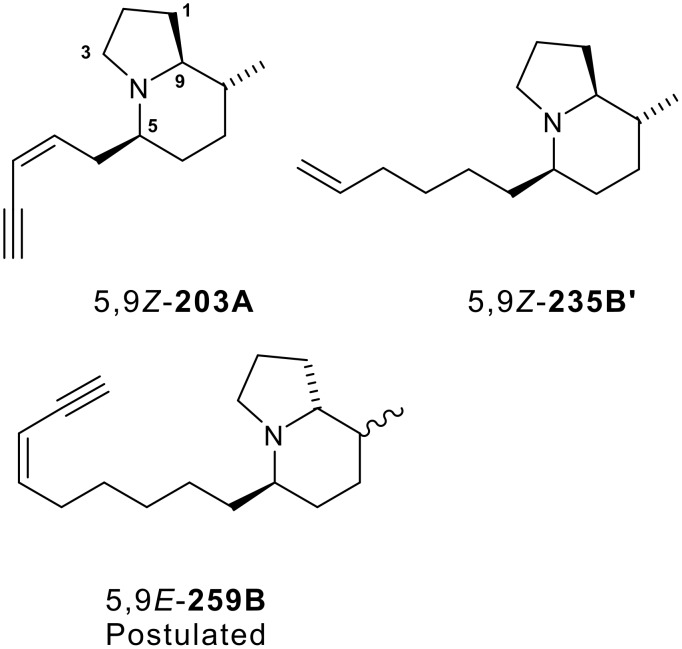
Subclasses of diastereoisomeric 5,8-disubstituted alkaloids. The absolute stereochemistry of 5,9*Z*-**203A** and 5,9*Z*-**235B'** are as shown, while the structure shown for 5,9*E*-**259B** is tentative as postulated based on mass and FTIR spectra [2]. Almost all of the 5,8-disubstituted indolizidines detected in frog skin extracts have proved to be the 5,9*Z* isomers [2].

Indolizidines are common in nature [1] and to date over eighty 5,8-disubstituted indolizidine alkaloids have been isolated from the skins of frogs [2]. Due to the scarcity of such indolizidine alkaloids from the natural sources, for the most part the biological properties of these materials have not been fully evaluated. However, synthetic 5,8-disubstituted indolizidine 5,9*Z*-**235B'** (Figure 1), has recently been shown to be a potent and selective non-competitive inhibitor of nicotinic acetylcholine receptors [3]. Earlier work had reported that indolizidines 5,9Z-**203A** and 5,9*Z*-**235B'** (Figure 1), and other 5,8-disubstituted indolizidines were non-competitive blockers of the ganglionic subtype of nicotinic receptors [4]. For most of the 5,8-disubstituted indolizidines the structures have been assigned by a combination of GC-mass spectrometry and GC-FTIR spectroscopy [2] and such structures must be considered tentative until NMR studies on isolated pure compounds can be obtained or until synthetic material is available for comparison. In the EI-mass spectrum of 5,8-disubstituted indolizidines loss of the C5 chain gives rise to the base peak, identifying the mass of the C5 substituent. The resulting cation undergoes a retro Diels-Alder fragmentation losing an alkene thus identifying the mass of the C8 substituent. Once the gross structure has been assigned, analysis of the vapor-phase infrared spectrum, particularly the Bohlmann bands, allows assignment of the relative configuration of the chiral centres at C5 and C9. When the two hydrogens on C5 and C9 are both axial (*trans* anti-parallel to the *N* lone pair), designated as 5,9*Z* (Figure 1), the presence of a strong, sharp Bohlmann band at approximately 2789 cm^−1^ confirms this relative configuration. In the alternative diastereoisomer when one hydrogen is axial and the other equatorial, designated as 5,9*E*, the Bohlmann band is weak and is shifted to 2810 cm^−1^. Most 5,8-disubstituted indolizidines detected in frog skin extracts have the 5,9*Z* relative configuration, with **259B** being very unusual in that it has the 5,9*E* relative configuration. Not surprisingly then, with the exception of the synthesis of two 5,9*E* diastereomers of the natural 5,9*Z*-**223V** [5], most of the synthetic effort has been directed towards the 5,9*Z* isomers and this has resulted in a large number of elegant approaches to these indolizidines [6–35].

## Results and Discussion

**Scheme 1:**
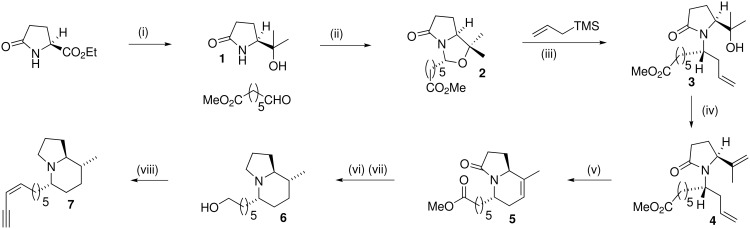
*Reagents:* (i) MeMgI. 96% (ii) PTSA 71%. (iii) TiCl_4_ CH_2_Cl_2_ 25 ^o^C 3d 68%. (iv) MsCl, Et_3_N, THF -40 ^o^C, 74%. (v) Grubbs' catalyst, 25 ^o^C CH_2_Cl_2,_ 90%. (vi) H_2_ MeOH Pt/C 79%. (vii) LiAlH_4,_ AlCl_3_, 73%. (viii) (a) Dess Martin periodinane, 77%. (b) Ph_3_P^+^CH_2_I I^-^, NaN(SiMe_3_)_2_ 51%. (c) TMS acetylene, CuI, Pd(Ph_3_P)_3_, Et_3_N then K_2_CO_3_ MeOH, 69%.

The absolute configurations of 5,9*Z*-**203A** and 5,9*Z*-**235B'** (Figure 1) and several other such 5,9*Z* indolizidines are known [2]. Thus, in analogy to such 5,9*Z* indolizidines it might be anticipated that for the 5,9*E* indolizidines the stereochemistry at C9 will also be *R*. We now report an enantioselective synthesis of the tentative structure postulated for ent-indolizidine 5,9*E***-259B**, which is outlined in Scheme 1 using (*S*)-pyroglutamic acid as the chiral starting material. The synthesis is extremely short, robust, does not utilise any protecting groups, appears to be completely diastereoselective at C5 and gives both diastereoisomers at C8.

Reaction of (*S*)-ethylpyroglutamate with an excess of methyl magnesium iodide gave the water soluble tertiary alcohol **1** in 96% yield. 7-Oxoheptanoic acid methyl ester was prepared by the literature procedure [36], by ozonolysis of 1-methoxycycloheptene, and then condensed with the amidoalcohol **1** with azeotropic removal of water to give the *N*,*O*-acetal **2** in 71% yield as a single diastereoisomer. It is likely that allylic strain of the lactam carbonyl group leads to the alkyl group preferentially occupying a pseudo-axial position [37–40]. Reaction of *N*,*O*-acetal **2** with trimethylallyl silane and titanium tetrachloride at room temperature for two days gave the product **3** in 68% yield. The alternate diastereoisomer could not be detected by NMR spectroscopy in the crude reaction mixture. Product **3** formally arises by attack of trimethylallyl silane from the least hindered face of the thermodynamically less stable *Z*-iminium ion and the mechanistic details of this intriguing transformation will be published elsewhere in due course. One-pot dehydration of the tertiary alcohol **3** was accomplished *via* the mesylate, and *in situ* elimination with triethylamine to give the diene **4** in 74% yield. Diene **4** smoothly underwent cyclisation to indolizidinone **5** when treated with Grubbs' first generation catalyst [41–42]. Analysis of the spectral properties of indolizidine **5** was considered convenient to confirm the stereochemistry at C5. It is known that in indolizidinones, with a carbonyl group at C3, the C5 hydrogen in the equatorial position will have an anomalously high chemical shift in NMR due to it lying in the deshielding cone of the lactam carbonyl group [5,37–38,43–44]. In the present case, the proton at C5 has a chemical shift at δ 4.24 ppm and the corresponding proton in similar indolizidinones with the 5,9*Z* relative configuration has a chemical shift at about δ 3.27 ppm. Reduction of the alkene **5** with hydrogen and a heterogeneous catalyst gave the product indolizidines as a mixture of C8 diastereoisomers. When platinum oxide was used as catalyst, a 1:1 mixture of diastereoisomers resulted, but when platinum-on-carbon was employed, a 4:1 mixture was produced with the isomer corresponding to** 6** (Scheme 1) predominating. We have previously shown [45], and there is also good literature precedent [46–47], that in indolizidines with unsaturation at C7-C8 there is a tendency for the addition reactions to occur on the concave face, although this obviously will be influenced by the presence of other substituents. In the present case, there is an additional axial substituent at C5, which again would encourage reaction from the concave face. Although the mixture of isomers proved inseparable at this stage, the relative configuration at C8 in both diastereoisomers could be readily assigned by examining the multiplets for the hydrogen at C9. For the major diastereoisomer the coupling constant *J*_8-9_ was 9.9 Hz, indicating a *trans* diaxial arrangement of these hydrogens and for the minor diastereoisomer the corresponding *J* value was 3.9 Hz. All that remained to complete the synthesis was the reduction of the lactam carbonyl group and the installation of the *cis*-enyne functionality. Simultaneous reduction of both the ester and the amide gave the alcohol **6**. Dess Martin oxidation [48] of the alcohol **6** gave an aldehyde, which on Stork Zhao reaction [49] gave the *Z*-vinyl iodide with a selectivity of 97:3. Finally, Sonogashira reaction [50] of the vinyl iodide with trimethylsilylacetylene followed by removal of the trimethylsilyl group gave synthetic **7**. At this stage the C8 diastereoisomers were separated by flash chromatography, though the minor component was contaminated with triphenyl phosphine / phosphine oxide residue from the Sonogashira reaction.

**Figure 2:**
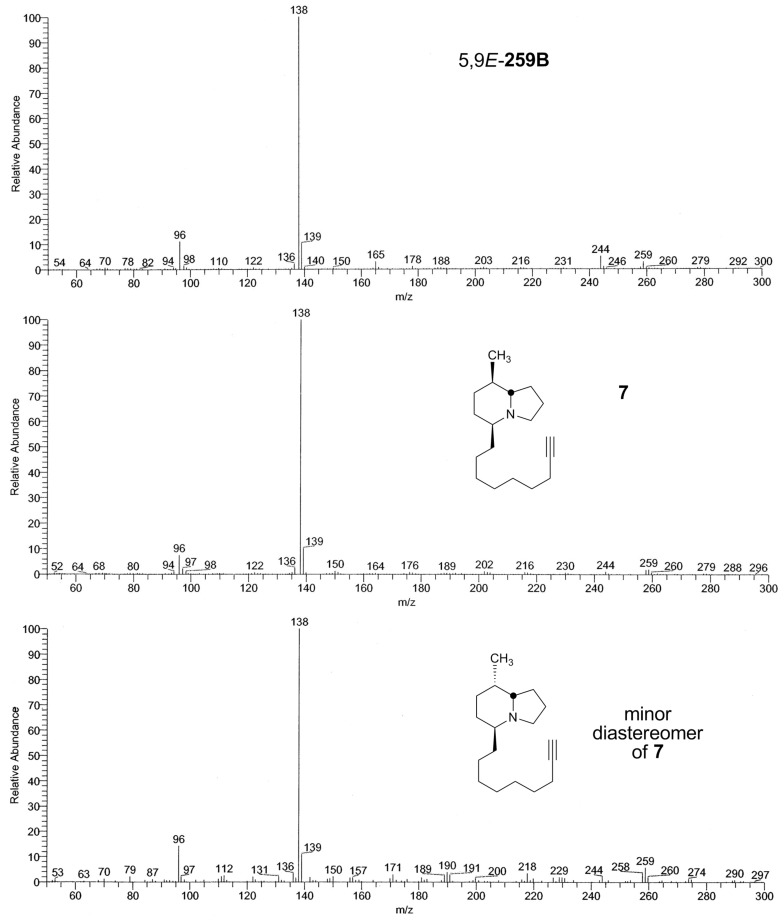
EIMS spectra of a) natural 5,9*E*-**259B**, b) synthetic **7**, and c) synthetic minor diastereomer of **7**. Structures are shown with relative configuration.

**Figure 3:**
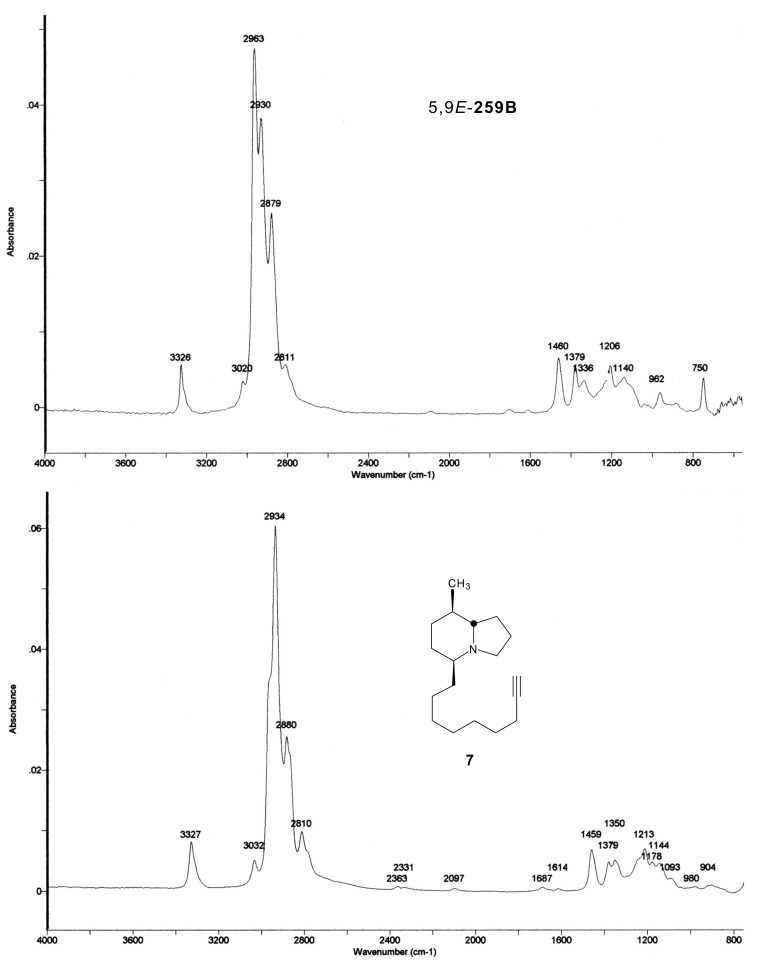
Vapor-phase FTIR spectra of a) natural 5,9*E*-**259B**, and b) synthetic **7**. Structure shown with relative configuration.

The two synthetic C8 diastereoisomers were compared to natural 5,9*E*-**259B** present in the alkaloid fraction obtained from a bufonid toad, *Melanophryniscus* *stelzneri* [51]. The GC mass spectra of the three compounds were very similar (Figure 2). However there was a greater loss of methyl for the natural alkaloid. The GC FTIR spectrum of the major synthetic diastereoisomer **7** differed from the natural 5,9*E*-**259B** in the finger-print region (Figure 3). In addition, the vinyl C-H stretching absorption band is at 3020 cm^−1^ rather than the expected 3032-3038 cm^−1^ for a conjugated CH=CH, as found in synthetic **7** and in the minor diastereomer. Finally, the intense C-H absorption band at 2963 cm^−1^ in natural 5,9*E*-**259B** suggests that two methyls rather than one are present. The corresponding band at 2961 cm^−1^ is merely a shoulder in the synthetic compounds that contain only one methyl. The GC FTIR spectrum of the minor synthetic isomer was very similar to that of the major isomer **7**, but due to a co-emerging contaminant the finger-print region could not be compared and the mixed FTIR is not shown. Remarkably, the GC retention time of the natural 5,9*E*-**259B** was markedly shorter than those of the two synthetic compounds as follows: Natural 5,9*E*-**259B:** 11.01 min; major synthetic isomer **7**: 13.01 min; minor synthetic isomer: 13.07 min. These retention times have been slightly adjusted to make them consistent with the retention times reported for the many frog skin alkaloids [2]. After hydrogenation the GC-retention times of the products (MW 265) were changed only slightly with the retention time of the perhydro-derivative of natural 5,9*E*-**259B** still markedly less than those of the perhydro-synthetics. This result proves that the carbon skeleton of **259B** is different to **7** and supports the proposal that there is a branch point in the C5 side-chain.

Clearly, a structural revision for 5,9*E*-**259B** is needed and it appears most likely that the point of difference is branching on the C5 side-chain. Isolation of 5,9*E*-**259B** for NMR spectral analysis will be required to establish the presence and nature of such branching. This hypothesis, if verified, is very significant because branching of the side-chains of 'izidine' alkaloids has been considered unlikely. The only documented case is the 5,6,8-trisubstituted indolizidine 5,9*E*-**249F**, isolated for NMR analysis from a dendrobatid frog, *Dendrobates auratus*, where there is an ethyl branch in the C5 substituent [51]. Further study will be needed to determine what other izidines detected in frog skin extracts have branch points in their side-chains. See Supporting Information File 1 for full experimental data.

## Conclusion

An extremely short entry to the unusual 5,8-disubstituted 5,9*E*-indolizidine alkaloids has been developed giving a synthetic sample of two possible structures corresponding to the structure postulated for indolizidine alkaloid 5,9*E*-**259B**. The synthetic compounds had mass and FTIR spectra similar, but not identical to those of the natural product, but the GC-retention times of the two synthetic C8 diastereomers, which were quite similar, differed markedly from that of the natural 5,9*E*-**259B**. Thus, the postulated structure of **259B** is not correct and further study will be required, in particular as to whether and where the side-chain at C5 is branched.

## Supporting Information

File 1 Experimental. Details of experimental procedures and data for characterisation of new compounds.
