# Supplementary material for: Facile synthesis of two diastereomeric indolizidines corresponding to the postulated structure of alkaloid 5,9E-259B from a Bufonid toad (Melanophryniscus)
Source: Beilstein J Org Chem. 2008 Jan 21;4:6. doi: 10.1186/1860-5397-4-6 (PMC2244622; doi:10.1186/1860-5397-4-6)
Supplement: File 1 — Experimental. Details of experimental procedures and data for characterisation of new compounds. [file Beilstein_J_Org_Chem-04-06-s001.doc]

Experimental

General

Melting points were recorded using a Kőfler hot stage apparatus and are uncorrected. IR spectra were recorded on a Perkin-Elmer Model 983G instrument coupled to a Perkin-Elmer 3700 Data Station as KBr disks, or films (liquids). Proton nuclear magnetic resonance (NMR) spectra were recorded at 300MHz and 500MHz using Bruker DPX 300 and DRX500 NMR spectrometers respectively. Chemical shifts are given in parts per million (δ) downfield from tetramethylsilane as internal standard and coupling constants are given in Hertz. Deuteriochloroform was used as solvent. Spectra splitting patterns are designated as s: singlet, d: doublet, t: triplet, q: quartet, m: multiplet and br: broad. Mass spectra were recorded using Double Focusing Triple Sector VG Auto Spec and accurate molecular masses were determined by the peak matching method using perfluorokerosene as standard reference and were accurate to within +/- 6 ppm. Optical rotations were determined using a Perkin-Elmer Precision Polarimeter model 241 using the specified solvent and concentration at the sodium D-line (589 nm) at ambient temperature. Analytical TLC was carried out on Merck Kielselgel 60_254_ plates and the spots visualised using a Hanovia Chromatolite UV lamp. Flash chromatography was effected using Merck Kielselgel 60 (230-400 mesh). A Finnegan-Thermoelectron GC-MS (GCQ or Polaris Q) was used to obtain mass specral data on natural and synthetic materials at NIH. The GC was fitted with a Restek RTX-5MS fused silica column, 30 m x 0.25 mm i.d. and used a temperature program of 100° C to 280° C at 10 ° C/ min. with a final hold time of 5 min. The injector temperature was 280° C. GC-FT
IR and EI-MS spectra were obtained with a Hewlett-Packard model 5890 gas chromatograph, having an HP-5 fused silica bonded capillary column (30 m x 0.32 mm i.d.) with the same temperature program as above. It was interfaced with a Hewlett-Packard model 5971 Mass Selective Detector and a model 5965B IRD with a narrow band detector. A Hewlett-Packard Chem Station was used to generate EIMS and FTIR spectra.

**(*S*)-5-(1-Hydroxy-1-methyethyl)-2-pyrrolidin-2-one (1).**

A solution of methylmagnesium iodide (130 mL, 2M) in diethyl ether was added via canula to a mechanically stirred, ice cooled, solution of (*S*)-pyroglutamic acid ethyl ester (10.02 g, 63.9 mmol) in THF (150 mL) and the resulting mixture was vigourously stirred for 6 hr. Saturated aqueous ammonium chloride solution (100 mL) was added with ice cooling and the mixture was stirred until all the solids dissolved. The organic layer was removed and discarded and the aqueous layer was placed in a continuous extractor and extracted with dichloromethane over 4 days. Each day the extraction solvent was replaced. The combined extracts were dried over sodium sulfate and concentrated to give the titled product as a white oily solid (8.83 g, 96%). For characterisation purposes a small sample was recrystallised from ethyl acetate/ether mixture to give the titled compound as a white amorphous solid, m.p. 62-64.5°C. For preparative purposes the crude material was used without purification in the next stage. $[\alpha]_{D}^{25}$** **= +14.9° (c = 0.105, CHC1_3_); (Found: C, 58.4; H, 9.2; N, 9.9 %. C_7_H_13_NO_2_ requires: C, 58.7; H, 9.1; N, 9.8 %); *m/z* (%) 144(MH^+^, 1.4), 128(9), 85(100), 84(73); *υ*_max_(KBr) 3403, 3333, 1684, 1379 cm^‑1^; δ_H_ (300 MHz) 1.137 and 1.219 (2 x 3H, 2 s), 1.80-2.00 and 2.00-2.19 (2 x 1H, 2m), 2.30-2.45 (2H, m), 3.56 (1H, dd, *J* = 8.0, 5.9), 4.18 (1H, br), 7.73 (1H, br); δ_C_ (75 MHz) 21.83, 23.15, 26.36, 30.48, 63.62, 71.59, 179.51.

**6-(3*R*, 7*aS*)-(1,1-Dimethy1,5-oxo-tetrahydropyrrolo[1,2-*c*]oxazol-3-yl)hexanoic acid methyl ester (2).**

*p*-Toluenesulfonic acid (0.08 g, 0.4 mmol) and 7-oxo-heptanoic acid methyl ester

(1.0 g, 6.3 mmol) were added to a solution of (*S*)-5-(1-hydroxy-l-methylethyl)-2-oxo-pyrrolidine in benzene (25 mL) and the resulting solution was heated, with removal of water, using a Dean-Stark trap for 18 h. The solution was cooled to room temperature, diluted with dichloromethane, washed with water, saturated sodium bicarbonate solution, dried over sodium sulfate and concentrated. Purification by flash chromatography (1:1 ethyl acetate:hexane) gave the titled compound as a pale yellow coloured oil (1.3 g, 71%). R_f_ = 0.26 (1:1 ethyl acetate:hexane); $[\alpha]_{D}^{25}$** **= + 30.9 (c = 0.2, CHC1_3_); Accurate mass measurement found MH^+^ 284.1859, C_15_H_26_NO_4_ requires MH^+^ 284.1862; *m/z* 284 (MH^+^ ,1), 253(12), 226(15), 154(70), 129(15), 124(39), 115(89), 101(19), 87(56), 83(58), 73(54), 59(87), 31 (12); υ_max_ (KBr film/cm^-I^) 1194, 1243, 1374, 1436, 1703, 1737, 2939; δ_H_ (300 MHz) 1.01 (3H, s), 1.29 (3H, s), 1.36-1.43 (6H, m), 1.61-1.68 (3H, 2m), 2.29-2.34 (1H, m,), 2.35-2.37 (2H, t, *J* = 7.4), 2.5-2.77 (2H, 2m**),** 3.66 (3H, s), 3.71 (1H, dd, *J* = 8.6, 4.1), 5.12 (1H, t, *J* = 5.3); δ_C_ (125 MHz) 14.55, 19.11, 23.91, 25.13, 25.21, 29.3, 34.34, 35.85, 51.76, 60.72, 67.54, 80.73, 85.69, 174.5, 177.6.

**(*S*)-7-[(*S*)-2-(1-Hydroxy-1-methyl-ethyl)-5-oxo-pyrrolidin-1-yl]-dec-9-enoic acid methyl ester (3).**

A solution of titanium tetrachloride (1.1 mL, 10.0 mmol), 6-(3*R*,7*aS*)-1,1-dimethy1,5-oxo-tetrahydropyrrolo[1,2-*c*]oxazol-3-yl)hexanoic acid methyl ester (**2**) (1.2 g, 4.0 mmol), allyltrimethylsilane (1.6 mL, 10.0 mmol) in anhydrous dichloromethane (10 mL), was stirred

at room temperature under a nitrogen atmosphere for 60 h. The solution was then diluted with dichloromethane (10 mL), washed with water, saturated sodium bicarbonate solution (10 mL), and again with water. The organic phase was dried over magnesium sulfate, concentrated, and flash chromatography (1:1 ethyl acetate:hexane) gave the titled compound as a pale yellow oil (0.9 g, 68%). R_f_ = 0.14 (1:1 ethyl acetate:hexane); $[\alpha]_{D}^{25}$ = +23.0 (c = 0.1, CHC1_3_); Accurate mass measurement found MH^+^ 326.2327, C_18_H_32_NO_4_ requires MH^+^ 326.2331; *m/z* 326 (MH^+^ 10), 284(6), 266(1), 126(2); *υ*_max_ (KBr film/cm^-1^) 915, 1173, 1240, 1436, 1686, 1738, 2938, δ_H_ (300MHz) 1.21 (3H, s), 1.26 (3H, s), 1.29-1.69 (10H, overlapping m), 2.05-2.32 (4H, overlapping m), 2.32-2.77 (2H, 2m), 3.37 (1H, dd, *J* = 7.3, 1.7), 3.5 (1H, m), 3.66 (3H, s), 5.01 (1H, d, *J* = 17.3), 5.03 (1H, d, *J* = 10.1), 5.76 (1H, m); δ_C_ (125 MHz) 22.66, 24.31, 25.15, 27.49, 28.69, 29.26, 32.03, 32.84, 34.36, 35.98, 51.86, 58.94, 70.45, 70.09, 117.44, 136.66, 174.27, 177.78.

**(*S*)-7((*S*)-2-Isopropenyl-5-oxo-pyrrolidin-1-y1) dec-9-enoic acid methyl ester** (**4).**

(*S*)-7-[(*S*)-2-(1-Hydroxy-1-methyl-ethyl)-5-oxo-pyrrolidin-1-yl]-dec-9-enoic acid methyl ester (**3**) (1.2 g, 4.7 mmol) in anhydrous dichloromethane (13 mL) was cooled to -10°C (ice/salt bath) under a nitrogen atmosphere and methanesulfonyl chloride (1.8 mL, 23.6 mmol) was added dropwise. After 5 minutes, triethylamine (6.6 mL, 47.2 mmol) was added and the resulting mixture was allowed to warm to room temperature and then stirred for 2 h. Water (10 mL) was added and the mixture was stirred for 24 h. The dichloromethane layer was separated, the aqueous layer extracted with dichloromethane (2 x 10 mL), and the combined extracts were washed with saturated sodium bicarbonate and water, dried over sodium sulfate and concentrated. Purification by flash chromatography (7:3 ethyl acetate:hexane) gave the titled compound as a pale yellow coloured oil (0.8 g, 74%). R_f_ = 0.4 (7:3 ethyl acetate:hexane); $[\alpha]_{D}^{25}$** **= +136.0 (c = 0.2, CHC1_3_); Accurate mass measurement found M^+^ 307.2138, C_18_H_29_NO_3_ requires M^+^ 307.2147; *m/z* 307 (M+, 16), 276(26), 266(30), 234(42), 206(5), 178(10), 164(7); *υ*_max_ (KBr film/cm^-1^) 913, 1174, 1321, 1419, 1437, 1684, 1736, 2936; δ_H_ (300 MHz) 1.21-1.33 + 1.59-1.73 (10H, overlapping muliplets), 1.71 (3H, s), 2.15­-2.18 (2H, m), 2.27-2.34 (2H, m), 2.48-2.51 (2H, m), 3.67 (3H, s), 3.68 (1H, t, *J* = 6.3), 4.04 (1H, dd, *J* = 8.7, 3.0), 4.88 (1H, s), 4.97 (1H, s), 5.01 (1H, d, *J* = 16.1), 5.04 (1H, d, J = 10.6), 5.71 (1H, m); δ_C_ (125 MHz) 17.10, 24.42, 24.86, 26.49, 28.93, 30.88, 30.95, 33.99, 37.28, 51.48, 53.59, 53.85, 114.22, 117.09, 135.96, 145.67, 174.17, 175.85.

**6-((5*S*, 9*S*)-8-Methy1-3-oxo-1,2,3,5,6,9-hexahydro-indolizidin-5-y1) hexanoic acid methyl ester (5).**

Grubbs’ catalyst (5.2 mg, 6.2 umol) was added in one portion to a solution of **(***S*)-7((*S*)-2-isopropenyl-5-oxo-pyrrolidin-1-y1) dec-9-enoic acid methyl ester (**4**) (0.04 g, 0.1 mmol) in anhydrous benzene (3 mL) under a nitrogen atmosphere and the mixture was allowed to stir at ambient temperature for 24 hours. The solution was concentrated and purification by flash chromatography (1:1 ethyl acetate:dichloromethane) gave the titled compound as a yellow oil (0.03g, 90%). R_f_ = 0.26 (1:1 ethyl acetate:dichloromethane); $[\alpha]_{D}^{25}$ = +3.0 (c = 0.1, CHC1_3_); Accurate mass measurement found M^+^ 279.1831, C_16_H_25_NO_3_ requires M^+^ 279.1834; *m/z* 279 (M^+^, 29), 248(8), 239(2), 238(3), 232(2), 219(2) 212(4), 164(11), 150(100), 83(6), 79(6), 59(11), 55(24); υ_max_ (KBr film/cm^-1^) 917, 1175, 1436, 1674, 1730, 2860, 2935; δ_H_ (300 MHz) 1.15-1.63 (12H, m), 1.66 (3H, s), 2.27-2.51 (4H, 2m), 3.66 (3H, s), 3.9 (1H, br m), 4.24 (1H, dt, *J* = 9.5, 6.4,), 5.37 (1H, m); δ_C_ (125 MHz) 15.26, 16.77, 26.57, 28.52, 29.69, 30.81, 33.92, 36.08, 44.65, 51.49, 58.56, 65.84, 118.16, 129.76, 174.26, 174.54.

**6-((5*S*, 9*S*)-8-Methyl-3-oxo-octahydro-indolizidin-5-yl) hexanoic acid methyl**

**ester**

A solution of 6-((5*S*,9*S*)-8-methy1-3-oxo-1,2,3,5,6,9-hexahydro-indolizidin-5-y1)-hexanoic acid methyl ester (0.6 g, 2.2 mmol) and 5% platinum-on-carbon (0.2g) in anhydrous methanol (60 mL) was hydrogenated on a Parr apparatus at 42 bar for 24 hours. The catalyst was removed by filtration through Hyflo Super Cel and the pad was washed with further methanol. Concentration gave the titled compound as an oil (0.5 g, 79%) and was a 4:1 mixture of diastereoisomers.

$[\alpha]_{D}^{25}$ = +67.0 (c = 0.1, CHC1_3_); Accurate mass measurement found M^+^ 281.2002, C_16_H_27_NO_3_ requires M^+^ 281.1991; *m/z* 281 (M^+^, 7), 250(5), 208(1), 152(100), 110(3), 84(5), 55(9); υ_max_ (KBr film/cm^-1^) 575, 733, 1376, 1685, 1738, 2860, 2934; δ_H_ (300 MHz) 0.90 (3H, d, *J* = 6.3), 1.28-1.64 (15H, m), 2.27-2.38 (3H, 3m), 3.06 (1H, dt, *J* = 9.9, 7.5), 3.67 (3H, s), 4.15 (1H, q, *J* = 6.8); The minor diastereoisomer shows a distinctive peak at 3.79 (1H, dt, *J* = 7.8, 3.9) which was used for assigning relative configuration and quantifying the mixture. δ_C_ (125 MHz) (major diastereoisomer) 17.88, 24.24, 25.16, 26.35, 27.88, 28.19, 29.38, 30.22, 30.88, 34.33, 39.45, 47.82, 51.81, 59.48, 173.89, 174.59.

**6-((5*S*, 9*S*)-8-Methyl-octahydro-indolizin-5-yl)hexan-1-ol (6).**

Aluminum trichloride (0.09 g, 0.7 mmol) in anhydrous ether (3.3 mL) was added to a suspension of lithium aluminum hydride (0.03 g, 0.7 mmol) in anhydrous ether (3.3 mL) and the mixture was stirred at room temperature for 20 min. A solution of methyl 6-((5*S*, 9*S*)-8-methyl-3-oxo-octahydro-indolizidin-5-y1) hexanoic acid methyl ester (0.10 g, 0.3 mmol) in anhydrous ether (6.5 mL) was added dropwise with effervescence and the mixture stirred at room temperature for 30 min. The reaction mixture was cooled to 0°C and saturated aqueous sodium hydroxide (1.8 mL) was then added and the mixture stirred for a further 30 min. at room temperature. The layers were separated and the aqueous layer extracted with dichloromethane. The combined organic extracts were washed with brine, dried over magnesium sulfate, concentrated and purified by flash chromatography (6:4 ethyl acetate:hexane) to give the titled compound (79 mg, 79 %) as an oil. R_f_ = 0.53 (6:4 ethyl acetate:hexane); $[\alpha]_{D}^{25}$ = + 9.0 (c = 0.2, CHCl_3_); Accurate mass measurement found M^+^ 239.2244, C_15_H_29_NO requires M^+^ 239.2249; *m/z* 239 (M^+^, 2), 184(5), 138(100), 96(13), 70(8), 55(6), 41(9); *υ*_max_ (KBr film/cm^-1^) 732, 1058, 1377, 1458, 2857, 2929, 3368; δ_H_ (300 MHz) 0.87 (3H, d, *J* = 6.5), 1.33-l.38 (10H, m), 1.56-1.97 (10H, m), 2.64 (1H, q, *J* = 8.5), 2.82 (1H, dd, *J* = 3.0), 2.95 (1H, m), 3.62 (2H, dt, *J* = 9.8, 6.6); δ_C_ (125 MHz) 19.15, 20.73, 25.90, 26.31, 26.78, 29.10, 29.56, 30.36, 32.88, 33.03, 36.11, 49.30, 56.22, 63.17, 63.33.

**6((5*S*, 9*S*)-8-Methyl-octahydro-indolizin-5-yl)hexanal**

Dess Martin periodinane (2.0 g, 0.5 mmol) was added to a solution of 6-((5*S*, 9*S*)-8-methyl-octahydro-indolizin-5-yl)-hexan-1-ol (**6**) (0.40 g, 1.5 mol) in anhydrous dichloromethane (10 mL) under a nitrogen atmosphere. The mixture was stirred at room temperature for 4.5 hours. Ether (50 mL) was added, giving a white precipitate which was poured into sodium hydroxide solution (1.3 M) (20 mL) and stirred for 10 min. The two layers were separated and the organic layer was washed again with a further portion of sodium hydroxide solution (15 mL) and then water. The combined organic extracts were dried with magnesium sulfate, concentrated and flash chromatography (85:15 dichloromethane:methanol) gave the titled compound (0.3 g, 77%). R_f_ = 0.55 (85:15 dichloromethane:methanol); $[\alpha]_{D}^{25}$** = **+13.0 (c = 0.2, CHCl_3_); Accurate mass found MH^+^ 238.2165, C_15_H_28_NO requires MH^+^ 238.2171; *m/z* 238 (M^+^, l), 180(2), 167(5), 152(2), 138(100), 96(9), 57(5), 55(7); *υ*_max_ (KBr film/cm^-1^) 735, 1378, 1459, 1667, 1726, 2858, 2930; δ_H_ (300MHz,) 0.87 (3H, d, *J* = 6.5), 1.3-1.8 (18H, m), 2.42 (2H, t, *J* = 7.3), 2.65 (1H, m), 2.85 (1H, m), 3.0 (1H, m), 9.8 (1H, t, *J* = 3.4).

**(5**S**, 9*S*)-5((*Z*)-7-Iodo-hept-6-enyl)-8-methyl-octahydroindolizine**

Sodium hexamethyldisilazide (0.3 mL) in dry THF (2 mL) was added to a suspension of iodomethyltriphenylphosphonium iodide (0.63 g, 1.2 mmol) in dry THF (4 mL) at room temperature. After stirring for 1 minute, the solution was cooled to -78°C and 6((5*S*, 9*S*)-8-methyl-octahydro-indolizin-5-yl)hexanal (0.2 g, 0.8 mmol) in THF (1 mL) was added dropwise. The reaction mixture was removed from the cooling bath, allowed to warm to room temperature and stirred for a further 1h. Dichloromethane was added and the mixture was washed with 2 M hydrochloric acid. The acidic solution was then basified using 2 M sodium hydroxide solution and extracted with dichloromethane. Purification by column chromatography (triethylamine:ether:hexane 1:10:50) gave the titled compound (0.3 g, 51%). R_f_ = 0.3 (triethylamine:ether:hexane 1:10:50); $[\alpha]_{D}^{25}$ = +15.0 (c = 0.2, CHCl_3_); Accurate mass found M^+^ 361.1269, C_16_H_28_N requires M^+^ 361.1267; *m/z* 361 (M^+^, 100), 346(100), 306(17), 235(6), 222(13), 208(22), 194(37), 180(17), 167(77), 166(81), 152(72), 151(61); *υ*_max_ (KBr film/cm^-1^) 668, 1146, 1384, 1458, 2855, 2928; δH (300 MHz) 0.85 (3H, d, *J* = 6.1), 1.18-2.16 (20H, m), 2.62 (lH, q, *J* = 7.9), 2.72 (1H, dd, *J* = 8.5, 3.6), 2.88 (1H, m), 6.22 (2H, m); δ_C_ (125 MHz) 19.35, 21.05, 22.52, 28.02, 28.30, 28.59, 29.47, 29.95, 34.94, 37.81, 49.40, 55.60, 61.96, 82.58, 82.67, 141.70.

**(5*S*, 9*S*)-8-Methyl-5((*Z*)-non-6-en-8-ynyl) octahydroindolizine (7).**

Copper (I) iodide (0.008 g, 40.0 mmol) was added to a solution of **(**5*S*, 9*S*)-5((*Z*)-7-iodo-hept-6-enyl)-8-methyl-octahydro-indolizine (0.10 g, 0.3 mmol), trimethylsilylacetylene (0.05 g, 0.5 mmol), triethylamine (0.07 g, 0.7 mmol) and Pd(P(Ph)_3_)_4_ (0.02 g, 0.01 mmol) in acetonitrile (5 mL) under nitrogen and the resulting dark brown mixture was heated at 40 °C for 20 min. The solvent was removed and the resulting residue dissolved in methanol (5 mL), anhydrous potassium carbonate (0.8 g) was added and this was stirred at room temperature for 1 hour. Ether (10 ml) was added and the reaction mixture was filtered through Hyflo Super Cel. Concentration gave a pale yellow oil which was purified by column chromatography (triethylamine:ethyl acetate 2:98) to give the titled compound (0.05 g, 69%) as one diastereoisomer. R_f_ = 0.21 (triethylamine:ethyl acetate 2:98); Accurate mass measurement found M^+^ 259.2301, C_18_H_29_N requires M^+^ 259.2300; *m/z* 259 (M^+^, 4), 204(9), 138(100), 96(14), 79(2), 65(4), 55(5); υ_max_(KBr film/cm^-1^) 1144 1213, 1379, 1459, 2816, 2880, 2934. 3032, 3327;. δ_H_ (300 MHz) 0.86 (3H, d, *J* = 6.3), 1.3-2.03 (20H, m), 2.32-2.36 (1H, q, *J* = 7.3), 2.78 (lH, dd, *J* = 8.8, 2.8), 2.95 (1H, m), 3.07 (lH, s, 5.44 (1H, d, *J* = 10.4), 5.9 (1H, dt, J= 10.8, 7.5);. δ_C_ (125MHz,) 18.9, 20.6, 22.2, 27.5, 27.7, 28.1, 28.6, 29.4, 29.5, 30.1, 37.2, 49.0, 55.1, 61.6, 80.4, 81.1, 108.1, 146.0.

The minor isomer was also isolated but was contaminated with triphenylphosphine/triphenyl phosphine oxide from the catalyst.

δ_H_ (300 MHz,) 0.94 (3H, d, *J* = 6.9), 1.25-1.76 (20H, m), 2.31 (1H, q, *J* = 7.3), 3.06 (1H, s), 3.25 (lH, m), 5.43 (1H, d, *J* = 10.8), 5.97 (1H, dt, *J* = 10.8, 7.5).
